# Developmental Dyslexia: Where Do We Go from Here?

**DOI:** 10.3390/brainsci10030151

**Published:** 2020-03-06

**Authors:** Paul E. Engelhardt

**Affiliations:** School of Psychology, University of East Anglia, Norwich NR4 7TJ, UK; P.Engelhardt@uea.ac.uk

**Keywords:** dyslexia, eye movements, reading impairment, temporal asynchrony, verbal efficiency hypothesis, reading interventions

## Abstract

This editorial follows an influential review paper published in Brain Sciences in 2018 (*What is Developmental Dyslexia?* by John Stein). In this editorial, I present a critical look at the arguments in Stein’s review, with a particular view towards “looking ahead”. In looking ahead, I will focus on why dyslexia has been largely neglected by psycholinguistics and, in particular, shortfalls in knowledge about sentence processing. I will highlight some things that I think psycholinguistic methodologies can contribute to the understanding of developmental dyslexia. The editorial will then turn to address the larger research context of dyslexia. In short, investigations of dyslexia tend to be conducted across a wide range of disciplines, and by individuals with varied backgrounds, divergent views, and different goals. One argument I advance is that dyslexia has reached a point where “interdisciplinary” collaboration is essential, and in the event that that is not successful, the field would at least benefit from “adversarial collaborations”. Finally, I briefly address the issue of interventions (raised by Stein) for older children and adolescents by returning to the contributions that psycholinguistics can provide to dyslexia. The crux of my argument here is that there exists a missing link in interventions, and that missing link is sentence-level language comprehension.

## 1. Stein’s Review—The Crux of the Argument(s)

The purpose of this editorial is to have a second look at the review article by Stein, which has gained a lot of interest and citations (32 by Google Scholar). The review has also had one Comment article by Blythe, Kirkby, and Liversedge [1], and then a Reply to the comment by the original author (Stein). I believe that the main argument in Stein’s original review is that “…the (phonological) theory is set at too high a cognitive level to be explanatory” (p. 1). In contrast, Stein pursues an explanation of dyslexia via magnocellular neuron deficiencies (magnocellular neurons are large transmission neurons found primarily in afferent visual ganglia, and projecting ultimately to the dorsal stream). These deficiencies result in temporal processing difficulties, including slower processing and an increased number of errors [2,3]. The strongest evidence for magnocellular deficits being a primary cause of dyslexia comes from studies showing that improvements (training) in magnocellular function improve reading abilities. Beyond that, Stein also reviews evidence connecting distinct wavelengths of light (yellow and blue) and the use of coloured lenses, which have been shown to help certain individuals with dyslexia read more efficiently.

To summarize the main argument(s) in the review, researchers should focus on the known brain abnormalities associated with dyslexia and what those abnormalities may suggest about the disorder. The main deficits primarily affect the visual modality, which affects eye movements and visual attention (see also, [4]). In addition, the focus is on temporal processing difficulty. I see Stein offering two caveats: the first is that he accepts that phonological theories of dyslexia are partially correct, and the second is that the auditory modality is affected in a similar way as the visual modality (resulting in temporal processing issues and increased errors). In short, phonological theories should take a backseat or secondary role in explanations of the disorder and instead, focus should return to the chronologically earlier theory of magnocellular impairment. Similarly, there is a very strong pushback against the conclusions about eye movement differences in dyslexia being the result of the disorder rather than the cause, which largely traces back to Rayner and colleagues (for a review, see [5]).

**Figure 1:**
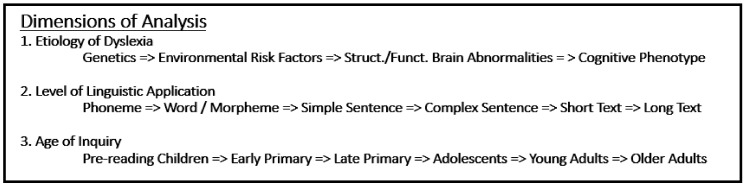
Multi-dimension overview of research in dyslexia, “levels of analysis” shown left-to-right within each dimension.

My opinion is that Stein’s review is quite limited and, to be fair, it was not intended to be exhaustive. Rather than take a critical perspective of the main ideas advanced by Stein, I feel that it is more productive to consider a more integrative and holistic view of research on dyslexia. Instead of arguing that we need to go back to earlier theories, I propose it is better to consider (1) where we are now and (2) the best way forward. To begin, I feel that it is important to be aware of several “dimensions of analysis”. Obviously, if the goal is to understand developmental dyslexia, then understanding the aetiology of the disorder is the primary goal (see dimension 1 in Figure 1). However, it is also very important to keep the level of linguistic application (dimension 2), as well as the age of the individuals with dyslexia in mind (dimension 3). Stein’s review very much focuses on the magnocellular hypothesis, as applied to word decoding in very young children. I would like to make two main points about dimensions 2 and 3. As discussed in more detail below, syntactic processing (sentence-level language comprehension) is very much under-studied (cf. [6,7,8]). Most eye movement studies looking at sentence comprehension focus on the processing of individual words within (sentence) contexts (e.g., [9,10,11]). With respect to age, there seems to be some competition between those focusing on children learning to read, which admittedly, has the most adverse effects on an individual (especially in terms of academic achievement), and those focusing on older age groups. Clearly, there are lifelong impairments with dyslexia, despite the majority of individuals with dyslexia ultimately learning to read, albeit not as efficiently [12]. Either the deficits (partially) remit or individuals gain compensatory strategies. Ultimately, individuals spend a great deal more of their life dealing with “adult-type” symptoms of dyslexia. Therefore, it is important that dyslexia is understood across the lifespan.

Returning to the arguments outlined in Stein’s review, he seems to contend that cognitive dysfunction models of dyslexia are problematic, and instead the better approach is to focus on brain abnormalities that “cause” dyslexia. I fully agree that with the assertion that causal models should cross levels of analysis, and so I take no issue with a starting point focused on the neurobiological level (although a geneticist may disagree). The next step in the process would be to explicate a model which describes the cognitive (and behavioural) levels of analysis. At this point, it is not clear to me the extent to which Stein’s approach implies (or assumes) neurobiological homogeneity, (I think it is clear that he does not advocate cognitive homogeneity). However, in the mid-2000s there was a quite noticeable shift in the thinking about developmental disorders and, specifically, multi-deficit approaches and/or the consideration of multiple developmental pathways [13,14]—that is, distinctive contributions to a disorder which should be dealt with in a unified manner, particularly in cases of comorbidity (e.g., ADHD). The success of these kinds of models depends heavily on largescale multivariate studies.

## 2. Psycholinguistics for Dyslexia—One Way Forward

My own training was in cognitive psychology and psycholinguistics, with an emphasis on parsing in adults and pragmatic issues around quantity of information. The field of psycholinguistics has traditionally been heavily focused on sentence-level language comprehension. Eye movements in reading features prominently, with consideration of various sorts of lexical variables, semantic manipulations, and temporarily ambiguous sentences. I believe that psycholinguistic methodologies and paradigms could be quite useful for dyslexia research, but the main one I advocate here is syntax. Syntactic processing refers to parsing and the process of determining meaning from groups of structured words. A reader must first decode individual words (lexical access), which involves retrieving word meanings from the lexicon. The parser then must assign words to grammatical roles and assemble a coherent syntactic and semantic representation. This will ultimately lead to propositional-level creation and the situation model that the sentence is describing. It is important to note that comprehension inferences happen incrementally in the course of processing a sentence.

There is very little research on eye movements in sentence-level language comprehension in dyslexia. I find this quite shocking. I am not sure why psycholinguists have not engaged more with dyslexia research. I’d estimate less than two dozen studies in the last two decades have examined eye movements in reading at the sentence level, using carefully constructed materials. What this ultimately means is that we know very little about incremental syntactic processing in dyslexia. Consistent with this, Stein’s review focuses exclusively on very young children (pre-school or early primary) and the processes associated with word decoding. There is perhaps one brief mention about how larger linguistic units may be impacted by magnocellular deficits. Like Blythe et al. [1], I also believe that the review is misleading in some aspects with respect to eye movements. I do not discuss the issues raised in the earlier commentary, but the review does give the impression that readers fixate letters individually, which may only be true in the very early stages of learning to read. I do not think ardent proponents of the dual-route model [15] or even those advocating that dyslexics tend to show problematic fixation locations nearer the beginnings of words would go as far as to suggest that readers fixate individual letters [16].

From my psycholinguistic and sentence-biased perspective, there was one assumption in the review that really stood out, although Stein made it implicitly. That assumption involves the temporal processing issues, and Stein even referenced synchronization. The *Synchronisation Hypothesis* [1,2] assumes that poor word decoding (1) adversely effects multi-word comprehension and (2) is due to a failure of automaticity. As a result, word decoding in individuals with dyslexia is a slow and time-consuming process, that requires substantially more cognitive effort. The Synchronisation Hypothesis, therefore, focuses on the timing in which information from bottom-up sources is provided to higher levels in order for comprehension to proceed efficiently. Individuals with dyslexia, in this case, experience asynchrony in language comprehension, which results in slowdowns and increased numbers of comprehension errors. Thus, one very nice possibility is that the neurobiological deficits highlighted by Stein may quite easily apply to sentence comprehension via synchronization. Another theory related to sentence-level comprehension is *Verbal Efficiency* [17], which also assumes that un-automated word decoding leads to excessive demands on cognitive resources and impedes/impairs higher-level (comprehension) processes. However, the focus here is on attention and working memory. Note that Stein specifically highlighted the role of visual attention in the review. However, working memory is not important for word decoding, as nothing must be held in memory, but working memory is required for sentence and text comprehension. Thus, there is a clear need to incorporate executive dysfunction(s) into multi-factor models of dyslexia and their role will be dependent upon dimensions of linguistic analysis and age [18,19]. Executive functioning tends to develop over a longer period of time than language.

### Reading Intervention

Before delving into the contributions that psycholinguistics may make to reading interventions, it is important to note that all of the dimensions/levels of analysis in Figure 1 can be investigated with respect to the deficits in dyslexia, but also in terms of the targets for intervention, with the one exception of genetics. Reading interventions differ depending on age. For younger children, interventions tend to focus on phonemic awareness, vocabulary, and fluency. Deficits in these areas can be partially overcome with efficacy reported across several meta-analyses [20] to increase reading performance by a half a standard deviation, mean effect size of ~0.40. In older children and adolescents, interventions tend to focus on the process of comprehension at the text level, and primarily involve the use of reading strategies, such as to think aloud by self-questioning and reflecting both during and after reading something. They are also expected to integrate what they read with existing knowledge in order to monitor understanding and process for meaning [21]. Intervention for *comprehension strategies* indicates small-to-moderate effects [22]. At present, there is no work bridging word-level interventions with text-level comprehension strategies. There is also no work looking at whether sentence-level interventions are effective in increasing syntactic processing (e.g., assigning nouns and verbs to their correct grammatical roles). Likewise, there is a need for studies to identify key pre- and post-intervention differences in eye movement signatures associated with skilled reading. Psycholinguists would presumably be invaluable in addressing all of these gaps.

## 3. Inter-Disciplinary Collison vs. Inter-Disciplinary Collaboration

In the review, Stein stated “…that dyslexia became the province of linguistic and educational psychologists rather than neurologists” (p. 2). As mentioned above, I have come to the field of dyslexia recently, and my opinions may be somewhat naïve. I find the literature quite fragmented and scattered, both in terms of where the research is conducted and where it is published. I disagree wholeheartedly with Stein about dyslexia being the province of “linguistic” psychologists, which I can only assume refers to “psycholinguists”. I also do not think it is the province of clinical psychology, unless comorbid disorders are involved (e.g., ADHD). Therefore, dyslexia would seem to be centrally a topic for educational psychology, and many of the journals specifically open to dyslexia studies are cross-indexed by educational psychology, communication studies, and special education (e.g., *Discourse Processes*, *Dyslexia*, *Scientific Studies of Reading*, *Journal of Research in Reading*). There are also a fair number of articles in neuroscience and experimental psychology journals, as well as multi-disciplinary journals (e.g., *Cognition*). In addition, research on coloured lens is most often published in ophthalmology journals, e.g., *Ophthalmic and Psychological Optics* and *Neuro-Ophthalmology and Visual Neuroscience*.

**Figure 2:**
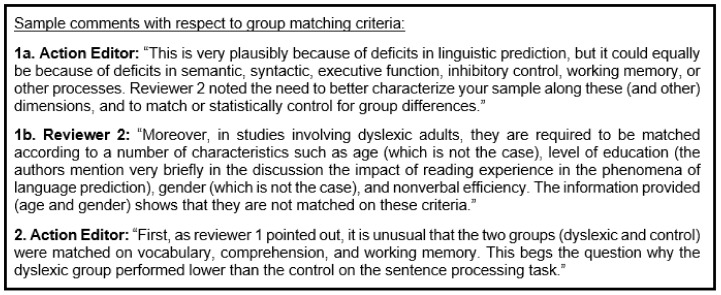
Sample comments from two action letters.

My experience as a cognitive psychologist trying to publish research on dyslexia has been quite difficult. For illustration purposes, I have included some sample comments from two action letters in Figure 2 below. My studies (conducted with a Phd student) focused on sentence comprehension in adults (mostly university students). In Point 1b, Reviewer 2 makes a strong point about the groups being matched, particularly on age and gender, and this was one of the main issues resulting in a recommendation of rejection. The action editor (Point 1a) does not actually mention age or gender, but instead includes a laundry list of possible individual differences measures. Three obvious points must be made about the action editor comment. The first is that the action editor does not say what the crucial variables to match on are. The second is that no study can include all possible measures. The third is that it does not matter if you match on all of these variables or you statistically control for these variables in inferential analyses; to do either would require hundreds, if not thousands of participants. Returning to the strong point made by Reviewer 2, our group differences in age were 19.7 years control vs. 21.7 years dyslexic, and gender was 14% male control vs. 41% male dyslexic. We did investigate these group differences. Age did not correlate with any of the dependent measures, as probably would be expected in young adults. Gender, in contrast, because the task primarily assessed reaction times, had more of an impact on the results. Specifically, females had faster reaction times and there were more females in the control group. However, any gender difference can be covaried/partialled and removed prior to considering differences with respect to dyslexia status. In a different paper, our groups (again, primarily university students) were matched on all of the individual difference measures we administered, but the action editor (Point 2) and one reviewer questioned why our groups did not follow commonly reported characteristics of dyslexia. The groups being matched on these variables was likely due to the fact that the dyslexic group (university students) were quite high-functioning. The more important point here is that the reviewer/action editor does not seem to grasp that if the groups are matched on key executive function and verbal intelligence measures, then the differences between groups must be due to sentence-level comprehension deficits.

I am not saying that all of my studies are perfect or that they all should be published in top-tier journals. The point of me including these comments is to show the kinds of roadblocks that have prevented me from getting this research out into the literature. These comments were the best examples in my action letters of clear contradiction. Clearly, some reviewers insist on matched groups (even in adults) and others seemingly protest against matched groups. It cannot go both ways. I am sure that others have worse examples of contradictory comments, but we all can appreciate the frustration. I am also aware that peer review is an imperfect process that becomes more strained every year.

### The Way Forward

**Figure 3:**
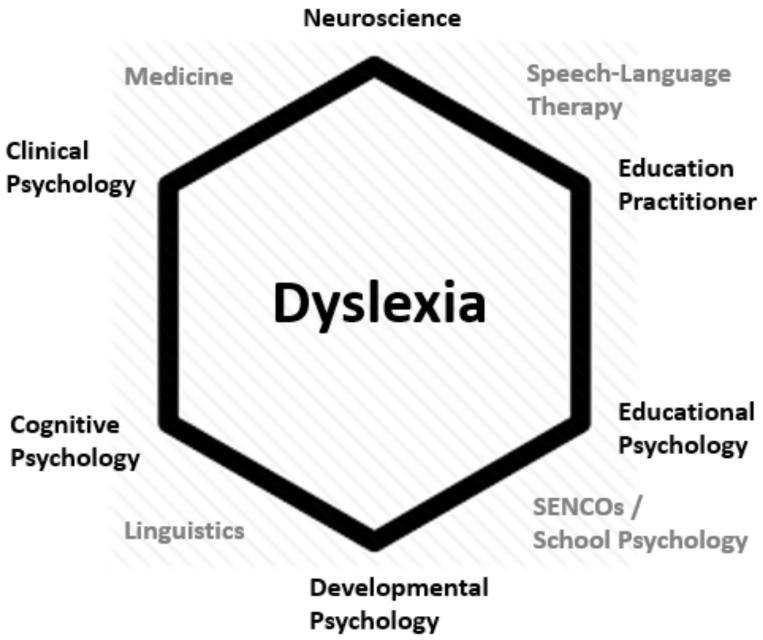
Interdisciplinary possibilities for dyslexia.

I do feel that there are some discipline-protective thinking patterns in the field of dyslexia research, and inter-disciplinary collaborations are one avenue past that. There is a very famous *Trends in Cognitive Science* paper [23], which uses a hexagon to describe interdisciplinary research. The idea was that each line running around and through the hexagon represented a unique area of investigation. I have re-created the figure below with respect to dyslexia (see Figure 3), and have intentionally left the lines out of the middle of this figure because they do not refer to unique areas of research (at least at this point in time). However, I find the figure useful in terms of judging the strength of research reports, and, perhaps more importantly, research proposals. The idea is that strength is inherent in the number of interconnecting lines within the figure (i.e., the interdisciplinary composition of the research team).

A second way forward might be more adversarial collaborations. Adversarial collaborations pit researchers who have competing views (or different approaches) against one another [24]. The resulting research is published in special issues, or within the same study, which allows for more direct comparisons of competing explanations or theories. Usually, in adversarial collaborations, there is a greater willingness to share stimuli and data. Rakow et al. [24] highlight two main benefits. The first is that it speeds up the process of getting research out. If a typical publication lag is 1–1.5 years, then there are possibly 3 years or more from the time an initial study is ran to the time in which the “counter” study would be published. This is glacial speed. The second benefit is that the person advocating the “losing” side in an adversarial collaboration will be more likely to change their mind/opinion/view/etc.

## 4. Conclusions

The goal of this editorial was to make several points with respect to the review paper *What is Developmental Dyslexia?* by John Stein. My own feeling is that that review is much too backwards-facing and too narrow (although Stein may disagree). Instead, what I have tried to do is take a much broader perspective (primarily incorporating psycholinguistics) and to put the focus on the future. I have also addressed some of my frustration at the publication issues I have encountered and doubt that I am alone in this. The most unfortunate outcome would be quality research going unpublished because of a “disciplines on collision” attitude. Perhaps some of the fault lies with me, in not engaging with researchers from other disciplines (i.e., doing what I recommend here).

From the most simplistic, pragmatic point of view, eye movements in reading are crucially important because students at universities in the UK on the disability register are given 25% more time in exams. For essays and research reports, students at our university put a “yellow sticker” on their work which is supposed to be taken into consideration in grading (i.e., not deducting for spelling and/or grammar). With respect to extra time, I am not aware of where the estimation of 25% comes from. Our eye-tracking studies have shown, for individual words within sentences (in adults), that 25% is probably about correct in terms of capturing total reading time differences between controls and dyslexics. However, when comprehension breaks down and re-reading is necessary, dyslexics show regression path durations that are approximately 50% greater than controls. Adolescents, which we have tested in much smaller numbers, have approximately 50% longer total reading times, and so 25% does not capture their difficulties. As is typical, there is much greater variability in reading times in samples of dyslexic readers compared to controls.

From a theoretical standpoint, moving forward, it is important to focus on multiple-deficit models, which cut across levels of analysis. Moreover, as mentioned by both Stein [25] and Blythe et al. [1], participant recruitment and samples sizes are an issue. In future, we need to consider increasingly largescale studies. Specifically, the need for multiple measures of candidate cognitive deficiencies, which permit latent variable modelling. It is also important to consider the deficits associated with dyslexia across the lifespan.

## References

[1] Blythe H.I., Kirkby J.A., Liversedge S.P. (2018). Comments on: “What is Developmental Dyslexia?” Brain Sci. 2018, 8, 26. The relationship between eye movements and reading Diffficulties. Brain Sci..

[2] Breznitz Z. (2003). The Synchronization Phenomenon. Fluency in Reading: Synchronization of Processes.

[3] Breznitz Z. (2006). Fluency in Reading: Synchronization of Processes.

[4] Prado C., Dubois M., Valdois S. (2007). The eye movements of dyslexic children during reading and visual search: Impact of the visual attention span. Vis. Res..

[5] Rayner K. (1998). Eye movements in reading and information processing: 20 years of research. Psychol. Bull..

[6] Rayner K. (1985). The Role of Eye Movements in Learning to Read and Reading Disability. Remedial Spec. Educ..

[7] Stella M., Engelhardt P.E. (2019). Syntactic ambiguity resolution in dyslexia: An examination of cognitive factors underlying eye movement differences and comprehension failures. Dyslexia.

[8] Wiseheart R., Altmann L.J.P., Park H., Lombardino L.J. (2009). Sentence comprehension in young adults with developmental dyslexia. Ann. Dyslexia.

[9] Hutzler F., Wimmer H. (2004). Eye movements of dyslexic children when reading in a regular orthography. Brain Lang..

[10] Hyönä J., Olson R.K. (1995). Eye fixation patterns among dyslexic and normal readers: Effects of word length and word frequency. J. Exp. Psychol. Learn. Memory Cogn..

[11] Jones M.W., Branigan H.P., Kelly M.L. (2009). Dyslexic and nondyslexic reading fluency: Rapid automatized naming and the importance of continuous lists. Psychon. Bull. Rev..

[12] Simmons F., Singleton C. (2000). The Reading Comprehension Abilities of Dyslexic Students in Higher Education. Dyslexia.

[13] Pennington B.F. (2006). From single to multiple deficit models of developmental disorders. Cognition.

[14] Sonuga-Barke E.J.S. (2005). Causal moedle of attention-deficit/hyperactivity disorder: From common simple deficits to multiple developmental pathways. Biol. Psychol..

[15] Coltheart M., Rastle K., Perry C., Langdon R., Ziegler J. (2001). DRC: A Dual Route Cascaded Model of Visual Word Recognition and Reading Aloud. Psychol. Rev..

[16] Hawelka S., Gagl B., Wimmer H. (2010). A dual-route perspective on eye movements of dyslexic readers. Cognition.

[17] Perfetti C.A., Hart L., Gorfien D. (2002). The lexical quality hypothesis. On the Consequences of Meaning Selection.

[18] Jeffries S., Everatt J. (2004). Working memory: Its role in dyslexia and other specific learning difficulties. Dyslexia.

[19] Perfetti C. (2007). Reading ability: Lexical quality to comprehension. Sci. Stud. Read..

[20] National Reading Panel (2000). Report of the National Reading Panel. Teaching Children to Read: An Evidence-Based Assessment of the Scientific Research Literature on Reading and its Implications for Reading Instructions (NIH Publication No. 00-4769).

[21] Beck I.L., McKeown M.G. (2006). Improving Comprehension with Questioning the Author: A Fresh and Expanded View of a Powerful Approach.

[22] Wanzek J., Wexler J., Vaughn S., Ciullo S. (2010). Reading intervention for struggling readers in the upper elementary grades: A synthesis of 20 years of research. Read. Writ..

[23] Miller G.A. (2004). The cognitive revolution: A historical perspective. Trends Cogn. Sci..

[24] Rakow T., Thompson V., Ball L., Markovits H. (2015). Rational and guidelines for empirical adversarial collation: A *Thinking and Reasoning* initiative. Think. Reason..

[25] Stein J. (2018). What is developmental dyslexia?. Brain Sci..

